# Providers’ Experience of Abortion Care: Protocol for a Scoping Review

**DOI:** 10.2196/35481

**Published:** 2022-02-02

**Authors:** B Dempsey, S Callaghan, M F Higgins

**Affiliations:** 1 Perinatal Research Centre University College Dublin National Maternity Hospital Dublin Ireland

**Keywords:** abortion, termination of pregnancy, reproductive health, health care providers, experience, scoping review

## Abstract

**Background:**

Despite being one of the most common gynecological procedures in the world, abortion care remains highly stigmatized. Internationally, providers have noted negative impacts related to their involvement in the services, and abortion care has been described as “dirty work.” Though much of the existing research focuses on the challenges of providing, many have also highlighted the positive aspects of working in abortion care. Despite the steadily increasing interest in this area over the past decade, however, no one has sought to systematically review the literature to date.

**Objective:**

The aim of this review is to systematically explore published studies on the experiences of abortion care providers to create a narrative review on the lived experience of providing abortion care, reflecting on what is already known and what areas require further exploration.

**Methods:**

This review will be conducted according to the framework outlined by Levac et al, which expanded on the popular Arksey and O’Malley framework. We will systematically search for peer-reviewed articles in 6 electronic databases: CINAHL, the Cochrane Library, EMBASE, PsycInfo, PubMed, and Web of Science. Following a pilot exercise, we devised a search strategy to identify relevant studies. In this protocol, we outline how citations will be assessed for eligibility and what information will be extracted from the included articles. We also highlight how this information will be combined in the review.

**Results:**

As of December 2021, at the time of writing, we have searched for articles in the electronic databases and identified 6624 unique citations. We intend to fully assess these citations for eligibility by the end of January 2022, chart and analyze data from the eligible citations by the end of March 2022, and submit a journal article for peer review by late spring 2022.

**Conclusions:**

The findings of this review will provide a comprehensive overview on the known experiences of providing abortion care. We also anticipate that the findings will identify aspects of care and experiences that are not reflected in the available literature. We will disseminate the results via a publication in a peer-reviewed academic journal and by presenting the findings at conferences in the areas of abortion care, obstetrics, and midwifery. As this review is a secondary analysis of published articles, ethical approval was not required.

**International Registered Report Identifier (IRRID):**

DERR1-10.2196/35481

## Introduction

“Dirty work,” first proposed by Everett Hughes, denotes professions that are socially, physically, and morally tainted [1]. Such professions may be described as “distasteful, disgusting, dangerous, demeaning, immoral, or contemptible,” while are also viewed as necessary by the society [2,3]. Common examples of such occupations may include garbage collectors, sex workers, and prison guards. Employees of these professions may be viewed as “dirty workers” and may experience stigmatization because of their association with their work. Carole Joffe was the first to describe abortion care as dirty work [4]. In the case of abortion, social taint may refer to the providers’ interaction with stigmatized individuals who seek care; physical taint may relate to the providers’ handling of fetal remains; and moral taint may relate to the ongoing debate regarding the unborn’s right to life. In the time since Joffe’s article [4], a growing body of literature has explored and reflected on the various challenges faced by providers of abortion care throughout the world, with common examples including the stigma ascribed to abortion care [5-8], the marginalization of abortion within mainstream medicine [9,10], and the difficulty of providing care under highly emotional circumstances [11,12].

Building on the early work of Everett, Ashforth and Kreiner created a theoretical model exploring how employees of stereotypically “dirty” professions may negate potential challenges and create positive social identities around their work [2,3]. They posit that a strong work group culture enables employees to reframe, refocus, and recalibrate aspects of their work—from stigmatized to socially important and noble. While providers may face challenges related to their abortion work, the literature has also highlighted the many positive aspects of working in abortion care, such as the benefits of strong support networks within the workplace and the pride taken in the work [13-15]. Given Ashforth and Kreiner’s model, these examples may help to negate or reduce the impact of the challenges that providers face, and they may help providers to bolster involvement and fulfillment in their work. As such, it is important to understand the existing literature to further this field of research. The aim of this review is to systematically explore research to curate a narrative and comprehensive review on the lived experiences of those who are directly involved in providing abortion care.

## Methods

### Study Design

In designing this review, various methodologies were considered. Given the broad nature of the research aim, a scoping review was considered the most appropriate [16,17]. This will allow us to examine the depth of the available literature in this area and to create a narrative review reflecting on what is known about the experience of providing care and what experiences or aspects of care are yet to be captured. To maintain rigor and replicability in our approach, we will follow the scoping review methodology outlined by Levac et al [18], which is an expansion of the widely cited Arksey and O’Malley framework [19]. This updated framework includes six stages, which are as follows: (1) identifying the research question; (2) identifying relevant studies; (3) study selection; (4) charting the data; (5) collating, summarizing, and charting the results; and (6) consultation. Each stage will be discussed in more detail.

### Stage 1: Identifying the Research Question

To begin, the research team must devise the research question, which will guide the review [18,19]. At this stage, it is essential to carefully consider the important aspects of the research area to revise and refine the research question. We did this by looking to relevant studies and reflecting on their findings. For example, the aim of the review at inception was to systematically explore and synthesize the known difficulties of providing abortion care (eg, stigma and increased risk of burnout). In consulting with the literature, however, it became clear that many studies in this area have also explored the positive aspects of providing abortion care, such as an increased sense of pride in one’s work and stronger collegial networks. For this reason, we rephrased the question to look at health care providers’ experience of providing abortion care, purposefully choosing the ambiguous term “experience” to elicit data on both the positive and negative aspects. From our reading, we are aware that examples of these experiences may include any positive or negative interactions within the providers’ societies, communities, or workplaces that are related to their abortion work, any aspects of care that are more emotionally or technically difficult to provide, any positive or negative emotions experienced during their abortion work, and any beliefs that the providers may hold about the services. The review will build on these examples and will identify other experiences, widening our understanding of the lived experience of providing abortion care. Additionally, we decided to use the broad term of “providing abortion care” to include any individual who is directly involved, clinically or nonclinically, in the care of patients who access the services. This iterative process led to the question, “what is the lived experience of individuals who are directly involved in the provision of comprehensive abortion care?”

### Stage 2: Identifying Relevant Articles

In stage 2 of the framework, the research team discussed and decided on the eligibility criteria, databases, and search strategy for the review.

#### Eligibility Criteria

The research team met twice to discuss and decide the criteria for identifying relevant studies. These meetings led to the following inclusion criteria: (1) original research articles published in peer-reviewed journals; (2) papers published in English; and (3) papers focused on the experiences of individuals who have direct patient contact with individuals accessing comprehensive abortion care services. In addition, the following exclusion criteria were also devised: (1) papers that are not original research (eg, editorials); (2) papers that are not published in a peer-reviewed journal; (3) papers on patients’ experiences of accessing abortion care; (4) papers on the technical or procedural aspects of providing care (eg, research on the efficacy of abortion medications); (5) papers on providers’ experience of managing “spontaneous abortion” (eg, miscarriage); and (6) papers on providers’ experience of managing postabortion care. No restrictions were set for the year, country, or reason for abortion.

#### Databases

To identify the citations, a systematic search was conducted in 6 electronic databases—CINAHL, Cochrane Library, Embase, PsycInfo, PubMed, and Web of Science.

#### Search Strategy

The search strategy was designed using the PCC (population, concept, and context) framework. The PCC framework has been recommended when conducting scoping reviews by the Joanna Briggs Institute (JBI) [16] and is regarded as a less restrictive version of the popular PICO (population or patient, intervention, control, and outcome) mnemonic, which is recommended for systematic reviews. The PCC mnemonic is also useful when searching for qualitative papers, which will be important for this review given the high number of qualitative studies in this area.

Following guidance from the JBI review manual, a 3-step iterative process was used to devise this search strategy [16]. Step 1 was to design a search string with basic terms, which we used in PsycInfo and PubMed to identify relevant citations. In step 2, we expanded this search string by including relevant keywords from the titles, abstracts, and keywords of citations that we found. This new search string was then used in all 6 electronic databases. We noted, however, that many of the identified citations explored patients and provider’s experience of spontaneous abortion, miscarriage, and ectopic pregnancy. As studies on these topics were unrelated to the review, we included an additional Boolean phrase to remove these papers. To test the validity of this third search string, we compiled a list of 32 articles that we knew would meet the eligibility criteria. We then used the string with the exclusion phrases in all 6 databases, and all 32 articles were successfully downloaded (Table 1). Step 3, the final step recommended by the JBI, is to search for unidentified papers in the reference list of the citations that have been included in the final review. We will also search for new articles published in the 6 electronic databases before we submit the review for publication, and we will search journals known to the research team, who have published research on the experiences of abortion providers. These journals include, but are not limited to, *Contraception*, *Reproductive Health Matters*, *PLOS One*, *International Journal of Gynecology and Obstetrics*, *Obstetrics and Gynecology*, *Reproductive Health*, *Women’s Health Issues*, *Family Planning Perspectives*, and *Social Science & Medicine*.

**Table 1:** PCC (population, concept, and context) elements for the study selection criteria, including an exclusion Boolean operator.

PCC^a^ element	Search string
Population	provider* OR “healthcare professional*” OR “health professional*” OR “healthcare worker*” OR “health worker*” OR Clinician* OR midwi* OR nurse* OR obstetric* OR gynaecolog* OR gynecolog* OR OBGYN OR physician* OR doctor* OR practitioner*
Concept	experienc* OR stigma* OR discrimin* OR prejudic* OR violenc*
Context	abortion* OR “termination of pregnan*”
Exclusion^b^	“spontaneous abortion” OR miscarriage* OR ectopic

^a^PCC: population, concept, and context.

^b^Though not included in the PCC framework, we added the “Exclusion” term when piloting the search strategy to reduce the large number of irrelevant citations.

### Stage 3: Study Selection

Stage 3 will be to search for articles. To begin, we will use the search string in the 6 electronic databases and download all the identified citations into an Endnote (Clarivate Analytics) library. We will then remove duplicates and conduct a title review on the unique citations that we find. For this stage, the lead author (BD) will meet with either coauthors (SC or MFH) to review titles as a pair, either agreeing on the inclusion or exclusion of citations or discussing before coming to an agreement. Once complete, all the citations deemed relevant will go through an abstract review. Here, BD will review all abstracts independently, and SC and MFH will each independently review 15% of the total citations. These independent screenings will be collated, and any discrepancies will be discussed as a group. Finally, a full text review will be conducted. Again, BD will independently conduct the full text review, and SC and MFH will both independently cross-examine 15% of the citations, discussing any discrepancies should they arise. This will leave the authors will a final list of citations to be included in the first draft of the scoping review.

### Stage 4: Charting the Data

In stage 4, once the relevant citations have been identified, information relevant to the review questions must be extracted and charted for use [18,19]. Following guidance from the JBI [16], a table will be created on Google Sheets for this purpose. Based on a preliminary exercise using the list of 32 research articles known to the review team, we developed 10 a priori categories to guide the charting of key findings. Article reference details and information on the study context and design will also be charted during this stage (Textbox 1). As suggested by Levac et al [18] and Daudt et al [20], BD will conduct a pilot before beginning the charting process and will chart information from 10 citations. The team will then meet after this exercise to discuss the inclusion of more key findings categories. This pilot phase will also be used by SC and MFH to give BD feedback on the information charted. Charting will be an iterative process; new information will be added to the table if needed, and the review team will hold meetings periodically to discuss progress. Once complete, SC and MFH will independently chart 15% of the citations, and these will be cross-examined with the chart created by BD. Any potential discrepancies will be discussed at a group meeting.

**Preliminary list of information to be charted from relevant articles by the research team. “Other” denotes our intention to create new categories during the data charting process if needed.**

Charting elements and characteristics of the study:

- Reference details
  - Article reference number (given to each article by the research team)
  - Study title
  - Authors
  - Year
  - Journal
  - DOI
- Study context
  - Aims or objectives
  - Country or region
  - Sample size
  - Job titles
  - Abortion procedures provided
  - The legal status of abortion care in each country
- Study design
  - Qualitative or quantitative or mixed methods
  - Data collection method
  - Sampling strategy
  - Analysis
- Key findings
  - Stigmatization of abortion within society
  - Abortion legislation
  - Challenges in providing care
  - Challenging interactions with patients
  - Challenging work group culture or interactions
  - Access to resources (eg, training, equipment, and space)
  - Negative personal impacts of providing care
  - Positive personal impacts of providing care
  - Personal beliefs about abortion
  - Positive work group culture or interactions
  - Other (specify)

### Stage 5: Collating, Summarizing, and Reporting the Results

As recommended by Levac et al [18], this stage will be conducted in 3 steps.

#### Steps 1 and 2: Collating and Summarizing

To describe the studies included in the review, tabular information will be collated in a Qualtrics form, which will be downloaded to SPSS (IBM Corp). Information collected by this form will include the article reference number and title, continent and country, number of providers, job titles of participants, and methodology (type of data, data collection methods, and analysis). A narrative summary of this information will also be included. As for information pertaining to the research question, we will conduct a thematic analysis following the guidance of Braun and Clarke [21,22].

#### Step 3: Reporting the Results

The reporting of the review findings will be informed by Ashforth and Kreiner’s model of “dirty work” [2,3]. This will be carried out by identifying the challenges that providers may experience because of their abortion work, the positive factors that may help them in this work, and finally, the providers’ own reflections on their involvement in the abortion care services. These broad themes will also explore the similarities and differences highlighted by the providers’ experiences in different countries, contemplating the impact that factors such as legislation, history, and religion may have on the providers’ experiences.

While not required by the scoping review methodology, a quality appraisal will also be conducted on the included studies using the Mixed Methods Appraisal Tool [23]. Studies deemed to be of low methodological quality will not be removed; rather, their low quality will be noted in the review. Each of the studies will be independently reviewed by 2 members of the research team (BD and SC), and any disagreements will be consolidated by the 3rd author (MFH).

### Stage 6: Consultation

As suggested by Levac et al [18], we intend to include the 6th stage of the framework, where the results of the scoping review are shared with experiential experts for feedback prior to publication. This review is one component of a larger academic research project to be completed by BD, and as such, meetings will take place within the Republic of Ireland to share the results of the scoping review, among other studies, with providers. In the future, other possibilities to consult international experiential experts will be considered.

## Results

As of December 2021, at the time of writing, we have searched the electronic databases and identified a total of 6624 unique citations. We intend to complete a title review, an abstract review, and a full text review on these citations by the end of January 2022. These reviews will be conducted to chart and analyze data from the eligible studies by the end of March 2022, and to prepare a journal article for peer review by late spring 2022.

## Discussion

### Principal Study Findings

The primary goal of this scoping review is to discover and map the existing evidence on the experiences of those involved in the provision of abortion care to understand the potential challenges and facilitators of providing care. This will act as a key point of reference for international providers, researchers, and advocates to further this area of research or discussion in their own territories, particularly in areas where they have recently or will in the future liberalize their abortion legislation. The review will also be relevant for health care workers who may need to reflect on what providing abortion care may involve before becoming involved in the services as well as offering those already involved in the services the opportunity to reflect on their practice. In conducting the review, we also predict that its findings will identify experiences that are lacking within the existing literature, highlighting new areas for exploration. It is also our hope that the findings can be used to inform the design of possible support interventions for providers, which may seek to minimize the impact of the various challenges of abortion work while bolstering the positive features. Thus, it is our hope that this review will be used to improve providers’ professional quality of life and job satisfaction and will help toward ensuring continued access to abortion care services around the world.

### Dissemination

The findings of this scoping review will be disseminated through a peer-reviewed publication in an international journal. The article will be reported in accordance with guidance from PRISMA-ScR (Preferred Reporting Items for Systematic reviews and Meta-Analyses extension for Scoping Reviews) [24]. The research team will endeavor to publish the review as open access to ensure that those interested internationally will be able to read its findings. We will also present the research at international conferences on abortion care, obstetrics, and midwifery. Finally, the consultation process, as outlined in “Stage 6: Consultation,” will help us to disseminate our findings with providers.

## Abbreviations

CINAHL: Cumulative Index to Nursing and Allied Health Literature
JBI: Joanna Briggs Institute
PCC: population, concept, and context
PICO: population or patient, intervention, comparison, and outcome
PRISMA-ScR: Preferred Reporting Items for Systematic reviews and Meta-Analysis extension for Scoping Reviews
